# Supplementary material for: Typical Sulfonamide Antibiotics Removal by Biochar-Amended River Coarse Sand during Groundwater Recharge
Source: Int J Environ Res Public Health. 2022 Dec 16;19(24):16957. doi: 10.3390/ijerph192416957 (PMC9779057; doi:10.3390/ijerph192416957)
Supplement: Supplementary file 1 [file ijerph-19-16957-s001.zip › ijerph-2018930-supplementary.pdf]

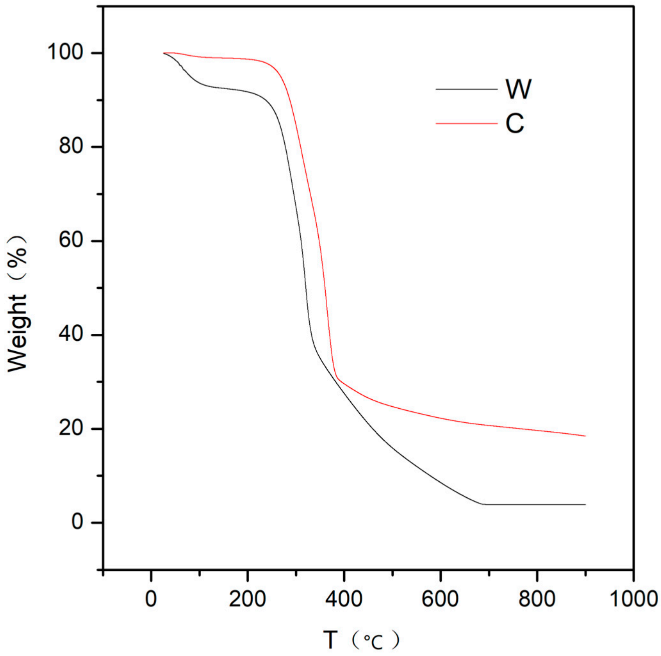

Figure S1 Thermogravimetric analysis of corn straw (C) and wheat straw (W)

Bromine ions did not adsorb on soil media during transport, so bromine ions were chosen as tracer ions to obtain the penetration curves in this study. The times ($t_{0.16}$, $t_{0.5}$, and $t_{0.84}$) corresponding to the concentration points C/C_0=0.16, C/C_0=0.5, and C/C_0=0.84 were obtained according to the penetration curves. D_L and V were calculated according to equations S1 and S2.

$$D_L = \frac{1}{8}\left(\frac{\mathrm{X-V}\,t_{0.16}}{\sqrt{t_{0.16}}} - \frac{\mathrm{X-V}\,t_{0.84}}{\sqrt{t_{0.84}}}\right)^2 \quad \text{(S1)}$$

$$\mathrm{V} = \mathrm{L}/t_{0.5} \quad \text{(S2)}$$

Where V is the flow rate, cm/min; L is the length of the soil column, cm; D_L is the dispersion coefficient.

Based on the penetration curve and the soil dispersion coefficient calculated by the above equations, the measured values and the actual calculated dispersion coefficients were brought into CXTFIT, and the CXTFIT software model was applied for parameter correction.

The theoretical retention time of pollutants was calculated according to equation S3.

$$R = \frac{T_{0.5\ SMX/TMP}}{T_{0.5\ Br}} \tag{S3}$$

Where $T_{0.5\ SMX/TMP}$ is the time at which sulfamethoxazole and methomyl was penetrated about half of the initial quantity; $T_{0.5\ Br}$ is the time at which bromine ion was penetrated about half of the initial concentration.

The vertical transport model of organic pollutants in unsaturated soil columns is based on the classical one-dimensional convection-dispersion equation (CDE).

$$R_d \frac{\partial C}{\partial t} = D_L \frac{\partial^2 C}{\partial t^2} - V \frac{\partial C}{\partial t} \tag{S4}$$

$$R_d = 1 + \frac{\rho_b}{\theta} K_d \tag{S5}$$

Where R_d is the retention factor; ρ_b is density of porous media, mg/cm^3; θ is volumetric water content, C is the concentration of contaminant, μg/L; D_L is hydrodynamic dispersion coefficient, cm^2/h; V is average pore water flow rate, cm/h; t is time, h; K_d is partition coefficient of solute between liquid and solid phases, L/kg.

In this study, we used CXTFIT software to fit the penetration curves obtained from recharged simulation experiments of SMX and TMP, solve the model parameters, and predict the concentration with time and space, use the equilibrium CDE model to fit the Br-ion penetration curves, and use the two-point nonequilibrium CDE model to fit and predict the distribution patterns of SMX and TMP in the soil column with time and space.

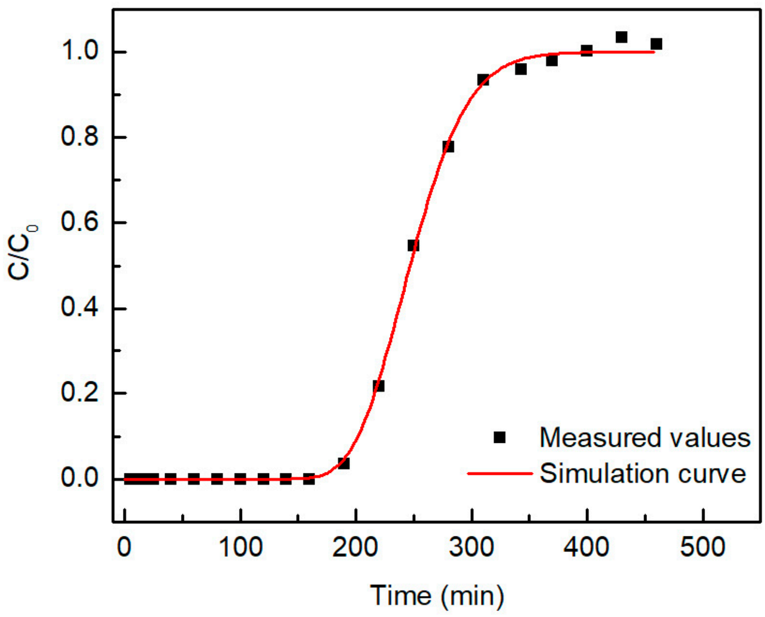

Figure S2 Penetration curve of bromine ion tracer

The adsorption amount of SMX in the column is calculated as:

$$\text{Adsorption amount} = \text{Input amount} - \text{Outflow amount} \quad \text{(S6)}$$

$$\text{Input amount} = \text{Solution concentration} \times \text{Input time} \times \text{Flow rate} \quad \text{(S7)}$$

Outflow amount is integrated according to the penetration curve by area.

High-performance liquid chromatographs (Waters 2695) equipped with photodiode array detectors (Waters 2998) were used for the detection of SMX and TMP. An XSelect®CSHTM-C18 (Waters, 5 μm, 4.6×150 mm) column was used. The mobile phase used were water with 0.1% trifluoroacetic acid (A), and methanol (B). The flow rate was 1 mL/min, the column temperature was 30 °C, and the retention time was 10.5 min. The detection wavelength was 269 nm, and the mobile phase was used in a gradient cycle, shown in Table S1.

Table S1 HPLC gradient elution procedure

Time (min)	Flow rate (mL/min)	%A	%B
1	1	70	30
2	1	55	45
5	1	55	45
5.5	1	70	30
10.5	1	70	30
